# Improving transportation networks: Effects of population structure and decision making policies

**DOI:** 10.1038/s41598-017-04892-2

**Published:** 2017-07-03

**Authors:** Federico Pablo-Martí, Angel Sánchez

**Affiliations:** 1 Grupo de Sistemas Complejos en Ciencias Sociales (SCCS), Departamento de Economía, Universidad de Alcalá, Alcalá de Henares, Madrid, Spain; 2 Grupo de Investigación de la Universidad Complutense de Madrid “Transporte, Infraestructura y Territorio (t-GIS)”, Universidad Complutense, Madrid, 28040 Spain; 3 Grupo Interdisciplinar de Sistemas Complejos, Departamento de Matemáticas, Universidad Carlos III de Madrid, 28911 Leganés, Madrid Spain; 4 Institute UC3M-BS of Financial Big Data, Universidad Carlos III de Madrid, 28903 Getafe, Spain; 5 Institute for Biocomputation and Physics of Complex Systems (BIFI), University of Zaragoza, 50018 Zaragoza, Spain

## Abstract

Transportation networks are one of the fundamental tools for human society to work, more so in our globalized world. The importance of a correct, efficient design of a transportation network for a given region or country cannot be overstated. We here study how network design is affected by the geography of the towns or nuclei to be connected, and also by the decision process necessary to choose which connections should be improved (in a generic sense) first. We begin by establishing that Delaunay networks provide an efficient starting point for the network design and at the same time allow us to introduce a computationally amenable model. Subsequent improvements lead to decentralized designs in geographies where towns are more or less homogeneously distributed, whereas radial designs arise when there is a core-periphery distribution of nodes. We also show that optimization of Delaunay networks outperforms that of complete networks at a lower cost, by allowing for a proper selection of the links to improve. In closing, we draw conclusions relevant to policy making applied to designing transportation networks and point our how our study can be useful to identify mechanisms relevant to the historical development of a region.

## Introduction

In spite of having received much attention in fields such as mathematics or sociology, particularly building on the pioneering work of Erdös and Renyi^1, 2^, the concept of complex network has become ubiquitous in many sciences and applied fields after the models introduced by Watts and Strogatz^3^ and by Barabasi and Albert^4^. The subsequent surge of activity, triggered by those two papers, has brought the physics’ community to lead the research effort in the study of networks, fostered by increased computing power and the availability of large databases of real networks^5^. Studies have focused on a huge variety of systems including transportation networks, phone call networks, the Internet and the World Wide Web, the actors’ coappearance network in movie databases, scientific coauthorship, citation and collaboration networks as well as systems of interest in biology and medicine, such as neural networks, ecological networks or genetic, metabolic and protein networks^6^.

During the first decade of the 2000s, the newly arrived science of complex networks has focused mainly on topological properties such as connectivity, clustering, centrality, community or modular structure, etc., trying to connect those features with the behavior of the systems under consideration and their dynamics. However, in recent years it has been realized that it is often necessary to consider the networked systems as being embedded in some underlying, possibly metric space^7^. One evident instance of this issue arises from spatial networks, where the constituent entities can be analyzed by forming a network in which links are defined by direct connections on the corresponding level of description. In that context, it is clear that the cost of establishing a new link is not the same for all cities, as it crucially depends on their distance, measured in the underlying space in between. Similarly, other properties of the network may depend on the metrics as well. It is worth mentioning that, in fact, quantitative geography has studied similar problems since almost 50 years ago^8^ and nowadays topics such as human mobility, design and planning of transportation networks, or city structure are being abundantly studied from interdisciplinary perspectives based on a spatial network approach^9, 10^.

Among spatial networks, transportation networks are particularly important, in so far as they govern key aspects of our society and are relevant to the understanding of many problems, such as disease spread, congestion, urban sprawl, and the structure of cities^11^. The viewpoint we adopt in this paper contributes to the research on network growth and, more specifically, on network improvement, seeing modifications to the network as the result of rational decisions by relevant actors in response to market conditions and policy initiatives^12, 13^. The question of network development and, in particular, its evolutionary nature, has been frequently overlooked by transportation planners and engineering, and understanding this issue is crucial given the enormous investments involved in this process. Indeed, effective investments and operations on infrastructure networks shapes the priorities for economic development of many regions and nations, and finding adequate plans and responses is a challenge for transportation policy-makers and professionals. It has been argued that top-down decision-making processes do not take properly into account the interconnected and interdependent nature of current transportation systems, and may lead to socially and economically undesirable outcomes. On the other hand, individual decisions and actions could eventually accumulate into development processes which are both path dependent and unpredictable. Our research intends to shed light on how different decision making procedures interplay with the geography and the pre-existing network and what are the corresponding outcomes at the level of the connected entities.

## Methods

**Figure 1:**
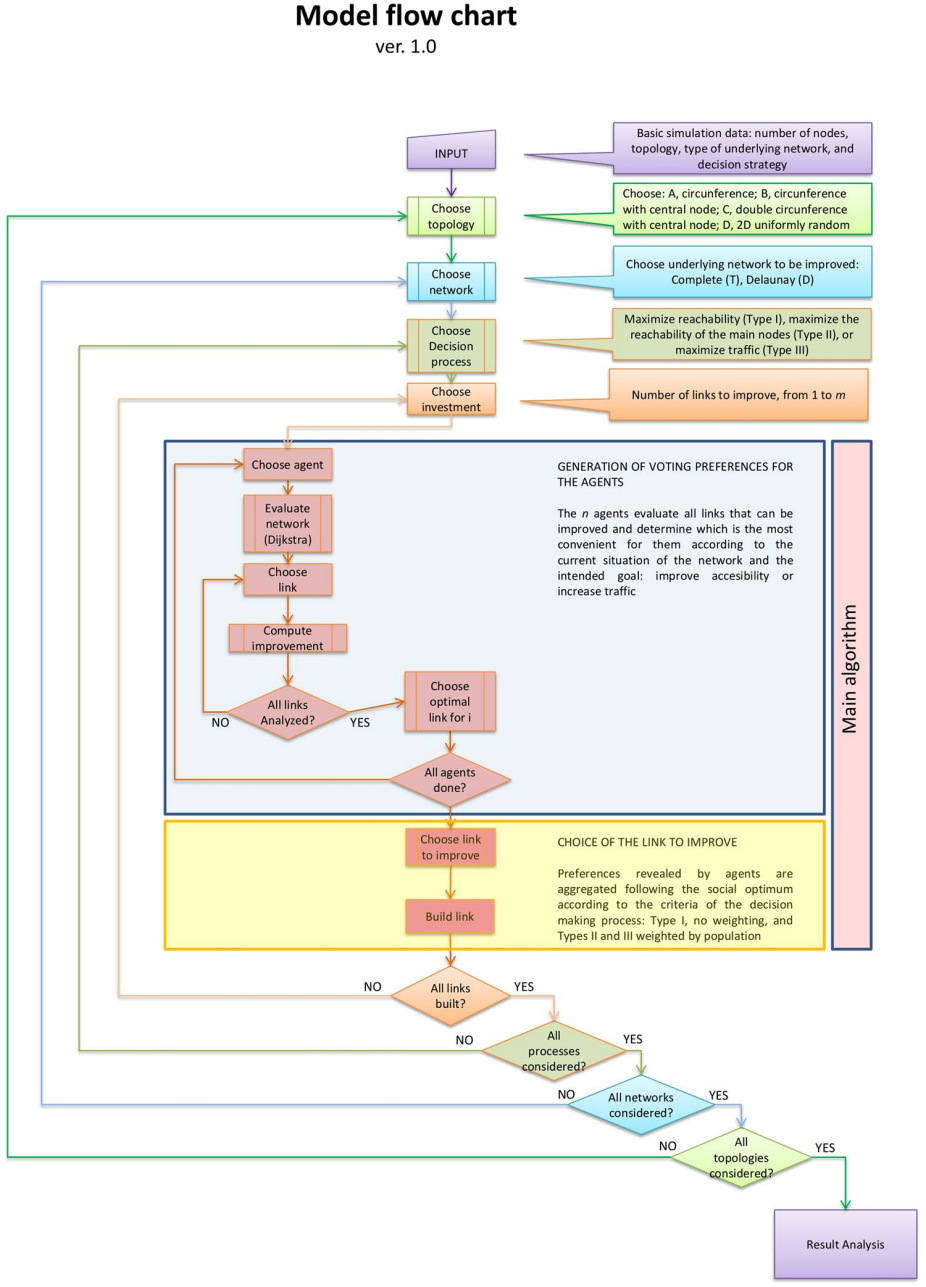
Flow chart of our model, showing the integration of the differerent features.

As stated above, the aim of this paper is to study how transportation networks evolve under different decision-making processes and starting from different geographies and types of connection network. We discuss below how we deal with each one of these aspects. For the convenience of the reader, we include in Fig. 1 a flow chart of our model so the integration of each of the aspects discussed below can be easily assessed.

### Geography

**Figure 2:**
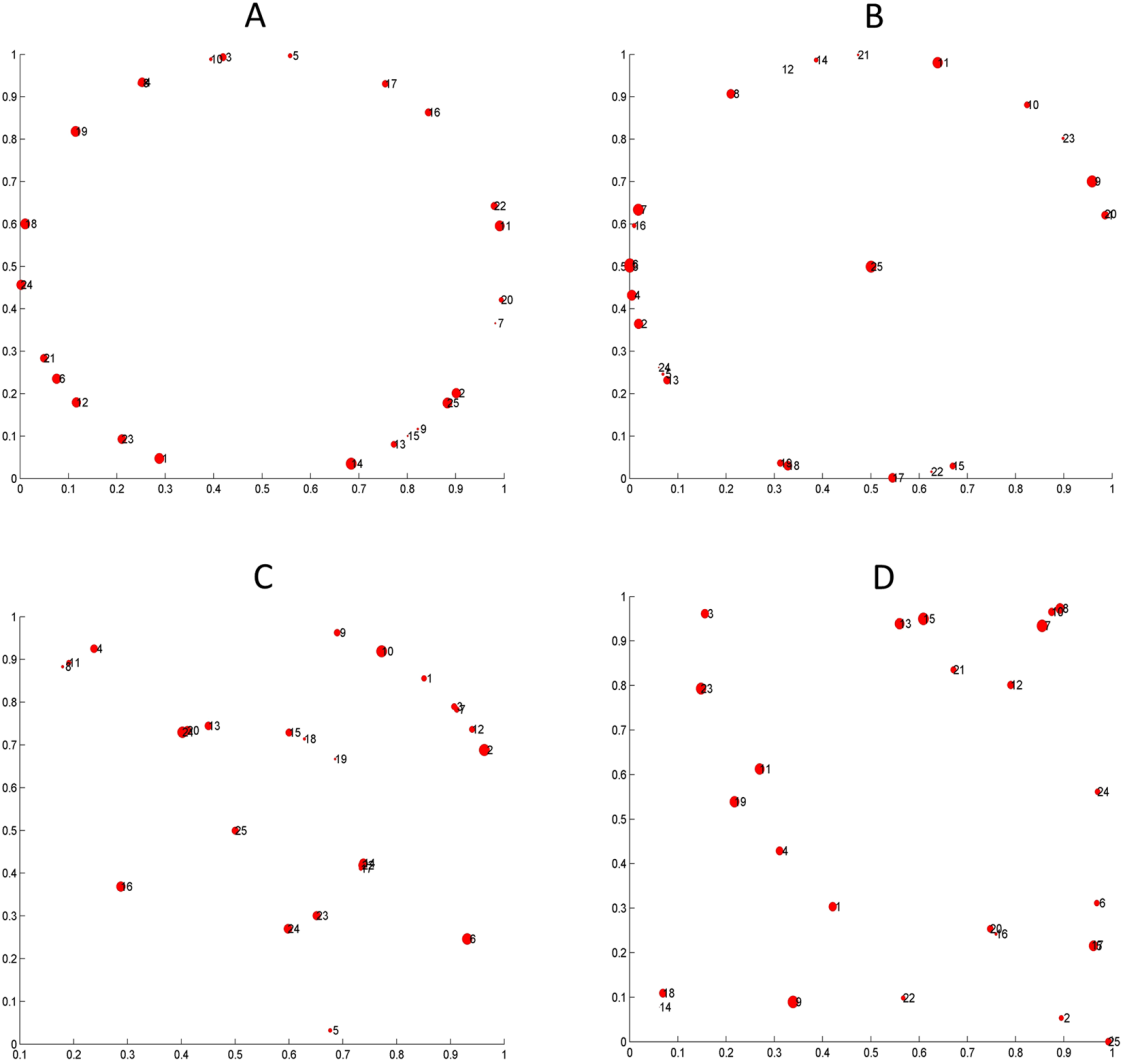
Representative examples of the different geographical distributions considered. (**A**) Circunference setup. This corresponds to the case of an island whose center is difficult to access, so communications take place along the coast such as, e.g., Iceland. (**B**) Circunference and central point setup. Regions with a large part of the population leaving in the periphery and in a central town. (**C**) Double circunference and central point setup. As in (**B**), but with a belt of intermediate towns between the center and the periphery, typically less populated thanthe others. (**B** and **C**) Exemplify countries such as France or Spain, while choosing (**B** or **C**) for the description depends on the relevance (actual or modeled) of the intermediate towns. (**D**) Random setup. Towns are uniformly distributed in the considered region, such as in, e.g., Germany, where such a distribution arises from a history of small kingdoms that became integrated only recently.

The first ingredient of our approach is a given geography of the region or country under consideration, understood as the positions of the population centers (towns, cities, villages) the network connects. To be sure, there is an infinite number of geographies one can consider, each with its own specific features and particular problems. However, in the spirit of addressing general problems, we focus on the differences between countries where the population concentrates in the periphery as compared with those in which the population is more uniformly distributed through the territory. Very roughly speaking, the former case would resemble a country like Spain or the United States, whereas the latter one would be similar to Germany, for instance. In addition, we will consider the effect of having a central hub and also the effect of having more or less towns. To this end we will use the following basic systems: a set of towns located on a circle, on two circles, with or without a town in a geographically centered position, or else a set of towns randomly scattered in the region under consideration. Examples of these basic configurations are shown on Fig. 2.

### Pre-existing network

As we have already stated, the purpose of our research is to understand how to improve an already existing transportation network. In this respect, it is important to note that this is a very general approach to the problem. Communication or transportation networks exist since many centuries ago. In ancient times, when the first societies started to appear, there were paths between neighboring villages, possibly used only by people on foot or on horseback, but they were bona fide paths. Later road or railway networks, from the Roman empire road network to the current highways, can then be considered as improvements on that first set of paths (notice that the new connection needs not lay exactly on top of the previous one, it should just link the same two cities). Therefore, we will look at how transportation networks change, in the understanding that this allows us to understand the status of the network at very many different moments in history just by changing the choice of pre-existing network.

With the goal stated in the previous paragraph in mind, once we adopt a specific geography from those presented in the previous subsection, we need to specify the current state of the connections among towns. In choosing models for those, we restricted ourselves to two connected networks: the complete network, i.e., there is a direct connection bewteen any two nodes (which may be more realistically associated to large towns connected by direct flights, but could in principle correspond to any mode of transport), and a Delaunay network. A Delaunay triangulation^14^ for a set of points in a plane is a connected network such that no point in the set is inside the circumcircle of any triangle in the network. In this way, the resulting network maximizes the minimum angle of all the angles of the network triangles, leading to less obtuse forms. It is interesting to note that the dual graph^15^ of the Delaunay triangulation is a Voronoi graph^16^. Delaunay networks are appropriate for the purposes of modeling a pre-existing transportation network, which in this case could be thought of as formed by roads or railroads, because another of its properties is that it contains the nearest neighbor graph as a subgraph, meaning that there are direct links from any town to its closest neighboring one. Furthermore, it can be seen^17^ that the maximum distance between two nodes following paths on a Delaunay network is at most twice the distance as the bird flies, while in actual geographical terms is much smaller, between 1.05 and 1.09^18^. To implement the Delaunay network, we used the built-in Matlab^19^ command delaunay. In the next section, we will see that, in fact, Delaunay networks are indeed a good representation of efficient transportation networks.

An additional comments is in order here regarding our choice of pre-existing networks. First, it could be argued that another suitable starting point would be a minimum spanning tree (MST)^20^. However, MSTs are not very good models for transportation networks because they lack redundancy by definition (trees have no cycles) and are highly inefficient in terms of total transportation costs. Although MST are extremely efficient in terms of construction costs, they are much less efficient in terms of transportation time, as it may well be the case that two nodes that are very close geographically end up having to travel a very long path to reach each other. A limited increase in redundancy, along with a few extra links, may lead to a substantial decrease in transportation time and to an increase in the network resilience against problems in any of its links, while very moderately increasing the constructions costs. On the other hand, MSTs are very sensitive to geographical changes: adding one node to the system or displacing one may lead to drastic changes in the new MST. Therefore, we do not consider this option here for its lack of applicability.

### Decision-making processes

In this work, we focus on the case in which decisions are taken by an external agent (government department, transport authority, etc) who tries to improve a pre-existing network by considering and balancing the interests of the different actors, in our case, the towns to be connected. It is important to understand this point because, as we will now define, decisions will be taken by an apparently “democratic” procedure, but this is not actual voting but rather the manner in which the external decision-maker weighs in the different characteristics of the town and the gains in network efficiency. Therefore, the alternative to our model would not be an external decision-making, but an external decision-making that uses other criteria for her choices (such as promoting a specific town, or a specific region).

In the above spirit, we complete our approach to the evolution of the transportation network by considering the case in which at every time step a particular link of the network is chosen and an investment is allocated to its improvement, leading to a reduction of the time needed to travel it by a factor of 75%. For the decision-making processes, i.e., for the procedure to choose links, we will restrict ourselves to bottom-up approaches (which, however, could also be seen as top-down if the decision-makers considered criteria based on the population distribution to choose the parts of the network to be improved) and, specifically, to the following ones, that touch upon different aspects of the weighting of towns and their main goal:

- Decision-making process Type I: Each town has one vote on the link to be improved. The aim of this rule is to reduce the total cost of travels. Connections have a weight proportional to travel time.
- Decision-making process Type II: Each town has one vote, weighted by its population, on the link to be improved. The aim here is to reduce the total cost in terms of the mean time spent by the population in traveling. Connections have a weight proportional to travel time.
- Decision-making process Type III: Each town has one vote, weighted by its population. The goal is different in this case, the rule attempts to increase total traffic. Traffic is determined by a gravitational model^21^ that is proportionally to the size of the connected nodes and inversely proportional to the square of the distance between them (measured in time). When travel time is decreased by the connection improvement, the traffic is increased. This procedure puts more weight on connections with relevant nodes, be it by proximity or by population.

In our model, the vote of each town is decided based on its own optimal choices, as determined by the minimal spanning tree originating from them. The minimal spanning tree for the towns is determined by using the Dijkstra algorithm^22, 23^.

## Results

### Efficiency: Delaunay triangulation vs complete networks

The first step in our research program is to establish the efficiency of Delaunay triangulations as an underlying transportation network among a set of cities. This is due to the fact that we are considering improving a pre-existing network, and there are a number of reasons why using a complete network as a starting point is less suitable. Indeed, the number of links agents must consider in order to find their optimal link to improve grows much faster in complete networks than in Delaunay networks, to the point that it becomes impractical or even impossible when the number is sufficiently large. In addition, if the number of links that can be improved is too large, the possibilities that the agents reach a consensus decrease accordingly. In fact, the existence of all one-to-one direct links favors a dispersion in the votes, leading very often to situations in which there are as many candidates for improvement as nodes. While this problem becomes less serious when new links are created, as nodes may prefer indirect paths that benefit from the improvements, it is is very sensitive to the initial configuration (path dependence) that is quite random due to the lack of consensus. Thus, in terms of modeling difficulties, it is clear that if the pre-existing network is a complete one, the required computing power is much larger, as all connections among towns are now eligible to be improved. In addition, in this case the variablity of the link length is much larger, and can include links whose improving cost is beyond the whole budget for improving the network. It is then clear that reaching any kind of consensus among the towns, to decide on which connection should be improved, would be almost impossible. This is not the case when using a triangulation as underlying network, as then most links are similar in length and improving cost. However, and more importantly, as we will now see, the complete network is also not a very efficient network in terms of construction costs and therefore it is not a good choice in our modeling procedure.

**Figure 3:**
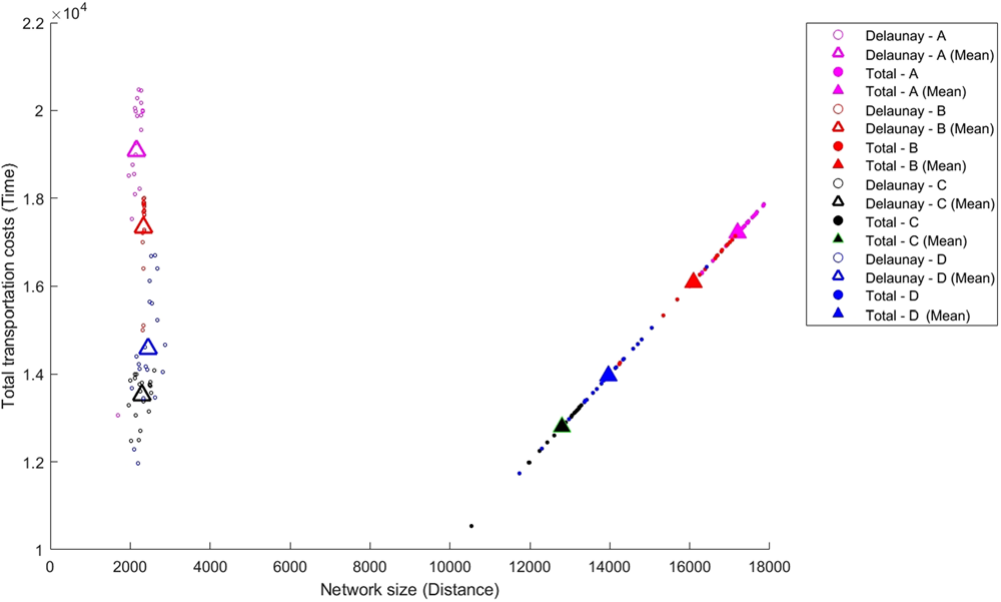
Total transportation costs for a region with 12 towns for different node distributions and preexisting connecting networks. Empty symbols: Delaunay triangulation. Filled symbols: Complete network. Node configurations: circunference (magenta), circunference with a center (red), double circunference with a center (black) and random (blue). Smaller symbols correspond to individual realizations, larger symbols to the mean for each case (preexisting network/geographical distribution).

In what follows, we will consider that a network is efficient if it provides a transportation service similar to that of a complete network, but at a lower construction cost, i.e., with less connections between cities. In this respect, prior to the improvement process, Delaunay networks are much more efficient than complete networks. Indeed, as can be seen in Fig. 3 the total costs of transport, measured in terms of the average time it takes to travel between two destinations, are somewhat larger for the Delaunay system, but they are strongly compensated by a much smaller construction cost (note that in the complete network all *n*(*n* − 1)/2 links have to be built). In a Delaunay network, its size depends basically on the number of nodes to connect and not on their specific geographical location, contrary to the situation in a complete network. In fact, Delaunay networks turn out to be more efficient because many links belong to many different trajectories.

**Figure 4:**
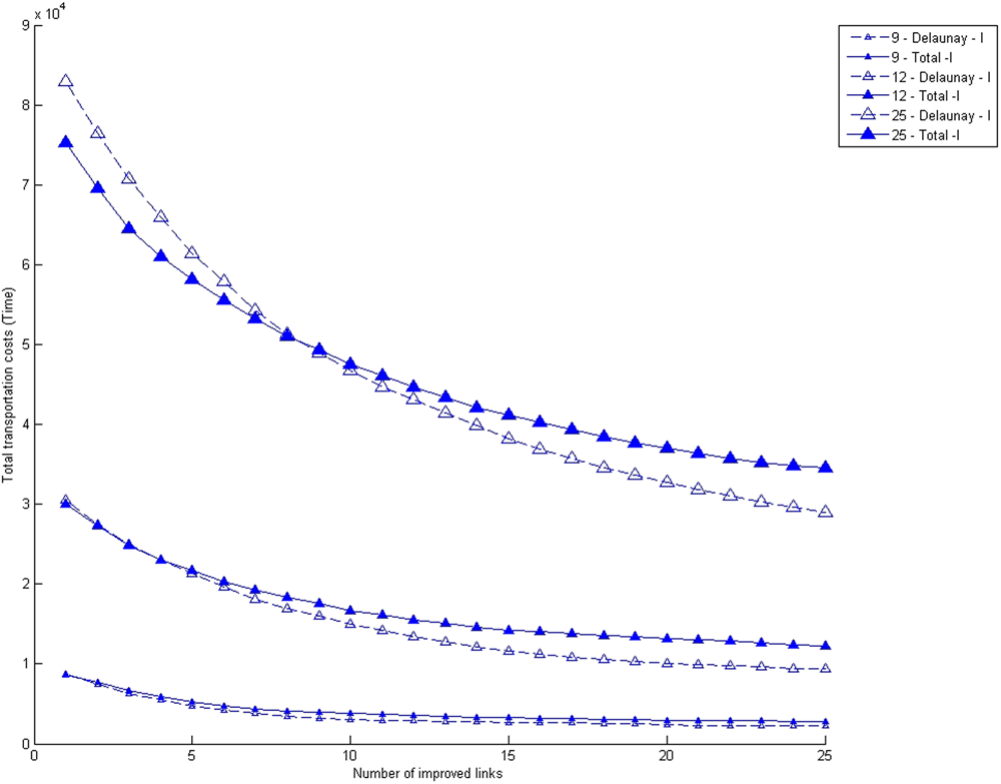
Total transportation costs for a region with 9 (lower lines, smaller symbols), 12 (middle lines, mid-size symbols) and 25 (top lines, larger symbols) towns for a uniform node distribution and the two types of connecting networks considered. Empty symbols: Delaunay triangulation. Filled symbols: Complete network. The decision-making process is of type I (one town, one vote).

When we now turn to the dynamic evolution of the transportation system by iterated single-link improvements, we find that complete networks only perform better for the first few modifications, but subsequently the co-utilization of links in the Delaunay networks mentioned earlier makes them perform better. Figure 4 shows that this is the case for different numbers of nodes, while we have found that their distribution does not lead to significant differences. Generally speaking, the improved links are shorter in the Delaunay network, leading to lower costs. On the other hand, it can be seen from the plot that total transportation costs for systems with 9 nodes are different than those of 12 and 25 nodes. The reason for this result is that the larger the number of nodes, the more links have to be improved for the Delaunay network to have more efficiency. It is clear that when no links are improved the complete network is necessarily more efficient than the Delaunay one, and subsequently plots always cross each other.

Of course, other triangulation schemes could have led to similar results. The fact that we have chosen the Delaunay algorithm in this work is due to a number of competitive advantages with respect to alternative triangulations. Thus, Delaunay triangles have their angles as similar as possible, implying more efficiency in so far as with a minimum number of links a larger surface is connected. This, along with the fact that the typical number of links a node has is about 6, implies in turn that angles are close to 60°. Interestingly, if one looks to the examples of France or Spain, there is a clearly visible hexagonal structure in the road system, highly resemblant of a structure generated by Delaunay process.

### Dynamics

**Figure 5:**
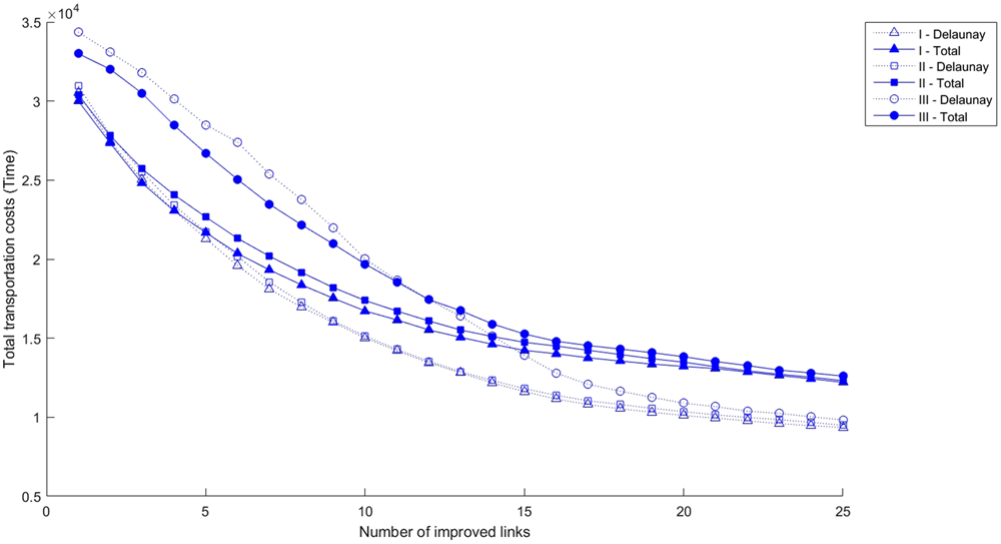
Total transportation costs for a region with 12 towns for a uniform node distribution, the two types of connecting networks considered and the three decision-making processes. Empty symbols: Delaunay triangulation. Filled symbols: Complete network. The decision-making process is of type I (one town, one vote; triangles), type II (weighted by population, squares) and type III (weighted by traffic, circles).

Let us now focus on the results from a more general viewpoint. Our thorough computational study allows us to look into the different factors shaping up transportation networks and assess their effects. Our starting point is the choice of decision-making process. As shown in Fig. 5, it turns out that for processes I and II the improvement of the transportation network leads to decreasing gains, while for process III we observe that the initial decrease is slower than in the other two cases, although the curve finally saturates in values around what arises from the other two decision-making algorithms. This occurs because process III leads to work on short links between close nodes that have a lot of traffic, while contributing less to global improvements. In all the geographies considered in the present work, the general result of the different decision-making processes is qualitatively the same.

**Figure 6:**
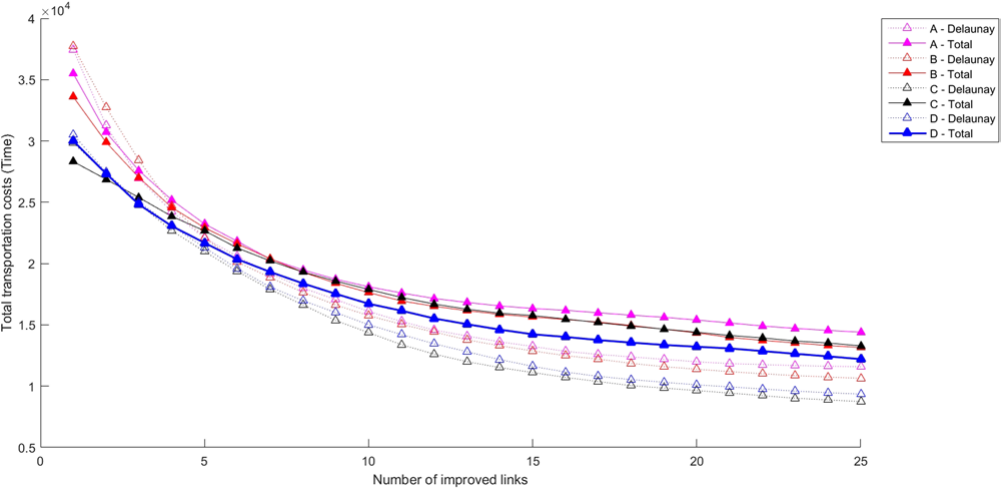
Total transportation costs for a region with 12 towns for the two types of connecting networks considered, the four geographical distributions and the three decision-making processes. Empty symbols: Delaunay triangulation. Filled symbols: Complete network. Node configurations: circunference (magenta), circunference with a center (red), double circunference with a center (black) and random (blue). The decision-making process is of type I (one town, one vote).

On the other hand, geography does have a clear effect on cost considerations. The transportation network shows lower costs for the case of uniform and double circumference maps, two situations that have very similar costs. Costs increase subsequently in the case of a circumference with a central node, while the single circumference turns out to be the most expensive as can be observed from Fig. 6.

**Figure 7:**
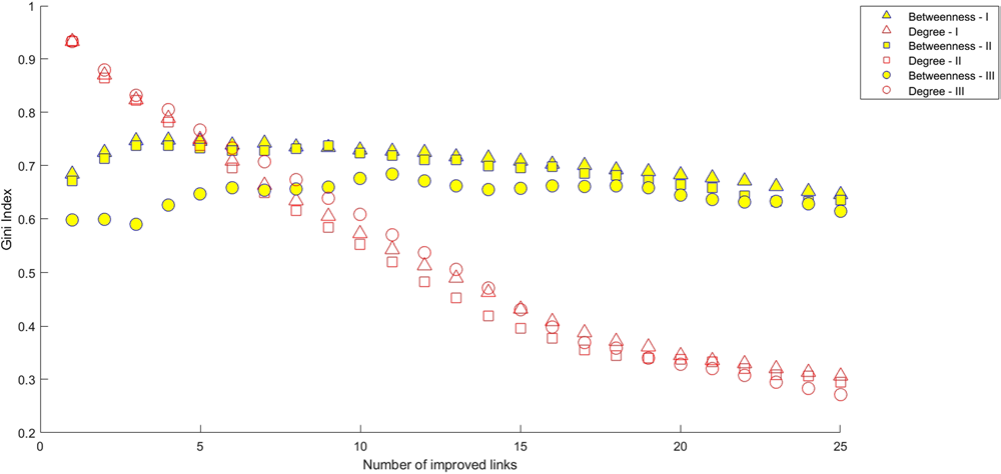
Inequality in the distribution of centralities in terms of the Gini index for a region with 12 towns with a uniform node distribution, the Delaunay triangulation connecting networks considered and the three decision-making processes. The decision-making process is of type I (one town, one vote; triangles), type II (weighted by population, squares) and type III (weighted by traffic, circles). Yellow: betweenness centrality; red, degree centrality.

Of course, the dynamical evolution of the transportation network arising from the sequential decision process we are analyzing here leads to changes in the centrality of the different nodes. In this respect, it is important to keep in mind that centrality is a non local property, in so far as it dependes on the position of the node in the network but also on the connections among other networks. Our approach in this paper allows us to look into this issue from a global viewpoint, accounting correctly for the changes in centrality and beyond what is usually the case in economic studies. In fact, most research carried out from the viewpoint of economics focuses on degree centrality, a local quantity, precisely because its locality makes it very visible for the general public; however, a more rigorous approach should be based on the betweenness centrality as intermediation effects are important for the whole network. To illustrate the different effects, Fig. 7 shows how centrality is distributed among the network nodes. The first improvement leads to a node with very large centrality, but as the process advances centrality becomes more and more equally distributed as measured by the Gini index of the centrality distribution. This phenomenon is observed for all three decision-making processes. If we now consider the betweenness centrality, the behavior is different: the values of the magnitude become less concentrated initially, but inequality increases after an intermediate number of improvements, for which the distribution of centrality is the most equitable one. In this case, decision-making processes of type III lead to oscillations in the Gini index which are not observed in the other two systems.

## Discussion

In this paper, we have presented a novel approach to understanding how transportation networks arise and can be improved by means of sequential decision processes. This is a much more realistic approach than those based on global optimizations, which usually lead to non local prescriptions involving several links that, on the other hand, may require resources beyond the budgets typically available to institutions to improve transport. Another difficulty of a global procedure (or a sequential procedure based on a complete network) is that in practice it would be almost impossible to make substantiated decisions as to which connections should be improved. Of course, it goes without saying that sequential processes have the drawback that they may lead to suboptimal behavior; however, the cost of globally optimizing a network involving many nodes makes the task prohibitively expensive even if restricted to Delaunay networks. In addition, failure to complete the designed network from a global viewpoint may lead to gross inefficiencies and equally suboptimal networks. Therefore, sequential decision processes are an important object of study. We stress that we are looking here at a specific decision procedure in which nodes vote one option only to improve in the next round. Having the option of voting for several options might have an effect (more on complete networks than on Delaunay ones) but we do not believe it is going to lead to qualitative changes and, in any event, it can be easily incorporated to the model. The results presented in the paper are examples supporting these conclusions, but we have carried out a much more thorough study involving all combinations of the ingredients of the model, other geographical distributions and perturbations of specific distributions by modifying the positions or the populations of one node. The results of this study are available in a dedicated website^24^ (where evolution is relevant, the plots in the website show movies of the network development as a function of time) and fully confirm the robustness of the conclusions above.

**Figure 8:**
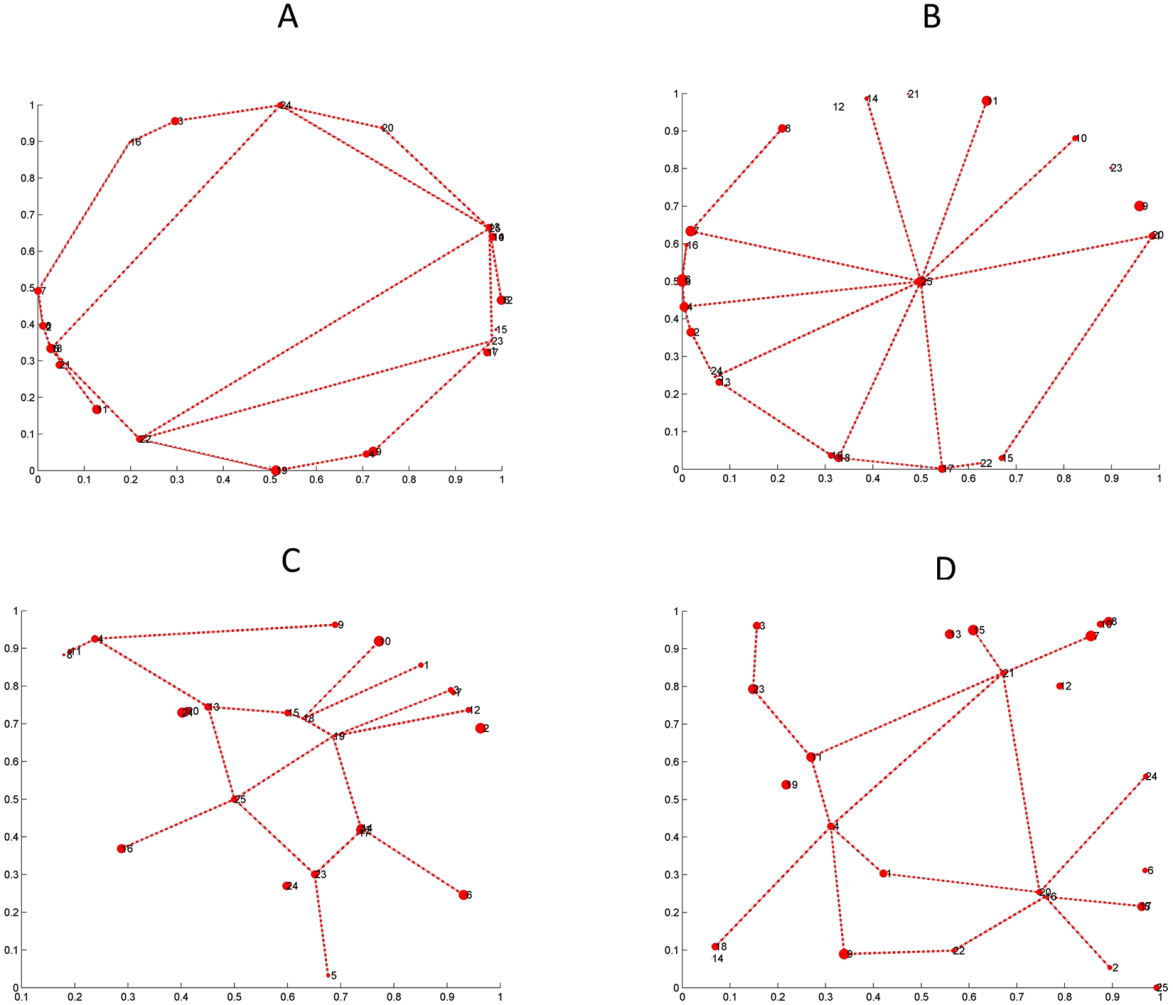
Snapshot of the network of improved connections among 15 towns after 18 iterations in the four geographical distributions considered starting from a Delaunay network and the type I decision process.

One of the most relevant conclusions of this work is that decentralized designs tend to be more efficient when the geography consists of homogeneously distributed nodes, while radial designs perform better when the important nodes are in the periphery without a specially relevant central node. Dynamically, a center-periphery layout tends to be more advantageous, although when many improvements are carried out the centralized and decentralized designs become more similar. It is interesting to realize that when decisions are decentralized, the lack of regional coordination may lead to absence of investments in densely populated regions, which calls for a multilevel decision process that incorporates the structure at different scales in an appropriate manner. Figure 8 illustrates this by showing an snapshot, after a sizable number of improvements have been made, for the four geographical distributions we are considering.

Distributed decision systems turn out to favor big investments, with large benefits in the form of long links that join different parts of the geography. Smaller investments with a high benefit/cost ratio but focused on a local scale are selected against, as agents value them according to the individual benefit and only a few agents have something to win from locally relevant links. It is also the case that when two important nodes are close in the geography they share investment decisions about far away links, while if there are more than two relevant nodes the competition among them may facilitate more drastic changes in the network improvement process. In any event, all these points make it clear that the discussion typically held in many countries about the convenience and efficiency of a centralized vs a decentralized transportation network is not the correct one to pose. Any consideration of this issue is highly conditioned by the budget available to improve the networks and by the geography, including population density. In this respect, another relevant conclusion of our work is that sequential improvement of a preexisting transportation network, based on efficiency criteria does not change much the most important centrality, the betweenness/intermediation one, and its effects concentrate on the more visible, local, degree centrality. This means that if the goal of an improvement policy is to equilibrate the differences between nodes in terms of their intermediation capabilities, the choices of the connections to work on should be guided by criteria different than pure efficiency and include this idea of achieving a level-playing field. The scale at which one looks at the problem is also an important point, and the optimal network for a given region changes drastically if it seen on its own or included at different levels in a country or a continent. We believe that the approach we have presented here, based on considering a pre-existing network well described by a Delaunay triangulation, may be very useful for policy-making consideration of different alternatives. Finally, it is interesting to note that our approach can also be very valuable in connection with historical research. When available data allow to track the time evolution of a transportation network (see, e.g., ref. 25), our model could be used to check which decision-making processes (the ones we are considering here or even others) are compatible with the historical development. We thus believe that this paper would stimulate further work using our tools in different fields and directions.
